# Assessment of the Functioning of Health and Wellness Centers in Kurud Block, Dhamtari District, Chhattisgarh: A Facility-Based Cross-Sectional Study

**DOI:** 10.7759/cureus.88071

**Published:** 2025-07-16

**Authors:** Veena G Dev, Manisha Ruikar

**Affiliations:** 1 Community and Family Medicine, All India Institute of Medical Sciences, Raipur, Raipur, IND

**Keywords:** ayushman bharat, health and wellness centers, human resources, infrastructure, teleconsultation

## Abstract

Background

Ayushman Bharat - Health and Wellness Centers (HWCs), now renamed as Ayushman Arogya Mandir, are envisioned to provide an expanded range of healthcare services. This study was conducted to assess the functioning of HWCs in the rural field practice area of the All India Institute of Medical Sciences, Raipur.

Materials and methods

This was a facility-based analytical cross-sectional study conducted in the Kurud block of Dhamtari District, Chhattisgarh. Half of the HWCs in the Kurud block were selected using stratified random sampling. Data on inputs and outputs from the selected 20 HWCs were collected using an observation checklist and analyzed using IBM SPSS Statistics for Windows, Version 21.0 (Released 2012; IBM Corp., Armonk, NY, USA).

Results

There was a significant shortfall (41.2%) in the availability of community health officers (CHOs). Most centers lacked adequate infrastructure, and none had the full complement of essential equipment and medicines. Only 17.6% of Sub-Health Center-HWCs (SHC-HWCs) and none of the primary health centers met the diagnostic standards outlined in the operational guidelines. Teleconsultation facilities were available in 70% of HWCs, and yoga sessions had been initiated in 85% of them. All HWCs had started screening for hypertension and diabetes. Based on performance grading, 52.9% of SHC-HWCs were classified as high-performing, while 47.1% were low-performing. Performance was found to be significantly associated with the presence of a CHO, availability of a laptop, webcam, and teleconsultation facilities.

Conclusions

To improve overall performance, all HWCs should address human resource gaps, ensure logistics align with operational guidelines, strengthen teleconsultation infrastructure - including uninterrupted internet access - and enhance information, education, and communication coverage and screening services.

## Introduction

The World Health Organization defines universal health coverage (UHC) as the ability of all individuals and communities to access the promotive, preventive, curative, rehabilitative, and palliative health services they need - of sufficient quality to be effective - without incurring financial hardship. In pursuit of UHC, the Government of India launched an ambitious healthcare initiative known as Ayushman Bharat. This program comprises two key components: the Pradhan Mantri Jan Arogya Yojana (PMJAY) and the Health and Wellness Centers (HWCs) [1].

In February 2018, the government announced plans to transform 150,000 existing sub-centers and primary health centers (PHCs) into HWCs, establishing them as foundational pillars of the Ayushman Bharat scheme [2]. Ayushman Bharat - HWCs (AB-HWCs), now renamed Ayushman Arogya Mandir, are designed to deliver a comprehensive range of services that address the primary healthcare needs of their catchment populations, ensuring accessibility, universality, and equity at the community level. These centers aim to provide a continuum of care across all stages of illness within the community. Their expanded service package builds upon existing PHC services and includes prevention, screening, and management of non-communicable diseases (NCDs); mental health services; basic ophthalmic and ENT care; oral healthcare; elderly care; and palliative care services [1,3,4].

Teleconsultation services at AB-HWCs connect patients to medical officers (MOs) and specialists, enhancing care quality, enabling remote support, and increasing patient engagement through virtual consultations. To further strengthen service delivery, outreach, and population-based screening, Performance Linked Payments have been introduced to incentivize the primary health care teams at the sub-health center (SHC) level [4,5]. According to the quarterly reports of Ayushman Bharat HWCs (April 2022), a total of 117,440 AB-HWCs had become functional by March 2022. During this period, more than 17.93 crore individuals were screened for hypertension and over 15.08 crore for diabetes. Additionally, 2.34 crore teleconsultations were conducted [6].

Chhattisgarh holds the distinction of hosting India’s first operational HWC - HWC Jangla in Bijapur - and was a key participant during the program’s initial implementation phase. As of July 31, 2024, a total of 5,826 Ayushman Bharat HWCs (including SHCs and PHCs) were operational across the state [7], reflecting Chhattisgarh’s strong commitment to the initiative. Despite this widespread implementation, there are currently no published studies assessing the functioning of HWCs in Chhattisgarh. Therefore, this study was undertaken to evaluate the performance and operational status of HWCs in the Kurud block of Dhamtari District, Chhattisgarh.

## Materials and methods

This was a facility-based analytical cross-sectional study conducted in the rural field practice area of the Department of Community and Family Medicine, All India Institute of Medical Sciences (AIIMS), Raipur, India, between May and June 2022. Ethical clearance was obtained from the Institutional Ethics Committee (AIIMSRPR/IEC/2021/945).

The Block Programme Manager and Block Medical Officer of Kurud Block were contacted to explain the purpose and methodology of the study. After obtaining permission, 20 HWCs - comprising 17 SHCs and three PHCs - were selected from the total list of 40 HWCs in the Kurud block through stratified random sampling. This represented 50% of all HWCs in the block, ensuring a manageable yet representative sample for robust analysis across different facility types.

For stratification, all 40 HWCs were divided into two groups based on distance from the community health center (CHC) Kurud: those located within 20 km and those beyond 20 km. Proportionally, 12 SHCs and 2 PHCs were randomly selected from the <20 km group, and 5 SHCs and 1 PHC from the >20 km group, using the RAND function in Microsoft Excel. This distance-based stratification aimed to reflect potential differences in accessibility and supervision from the CHC.

Each selected HWC was visited at least twice on weekdays (Monday to Saturday) during official working hours to ensure appropriate assessment of staff availability. All functional HWCs during the visits were included in the study. Healthcare providers were eligible for participation if they had been working at the HWC for more than six months and were present during the visit. Providers who did not give consent were excluded.

Each HWC was assessed for manpower, branding, infrastructure, availability of essential medicines and diagnostics, digitalization, use of telemedicine/IT platforms, biomedical waste management, data entry systems, health cards, family health folders, and services delivered, based on records and registers, using an observational checklist. A comprehensive pilot study was conducted prior to the main study to validate the tool and ensure feasibility. The pilot helped refine the checklist and confirmed its suitability for evaluating the predefined indicators.

Data were entered into Microsoft Excel (Microsoft Corporation, Redmond, WA, USA) and analyzed using IBM SPSS Statistics for Windows, Version 21.0 (Released 2012; IBM Corp., Armonk, NY, USA). Descriptive statistics were used to summarize the key inputs and outputs of the HWCs under the Ayushman Bharat Programme, including frequencies and graphical representations.

The performance of SHC-HWCs was evaluated using the indicators and scoring system from the Ayushman Bharat Program (Appendix A). Individual grades (A to E) were assigned based on this system. For analytical clarity, centers were grouped into two categories: high-performing (Grades A and B) and low-performing (Grades C, D, and E). A cutoff score of 75 was used, aligning with the minimum benchmark for Grade B, to ensure consistency with program-defined standards and facilitate meaningful comparisons.

Fisher’s exact test was used to assess the statistical significance of associations between center performance and various input and output indicators for SHC-HWCs.

## Results

Among the 20 study facilities, half (10; 50%) of the health centers were converted to HWCs during 2018-2019. This was followed by three centers (15%) in 2019-2020, six centers (30%) in 2020-2021, and one center (5%) in 2021-2022. All HWCs were found to be functional at the time of the visit.

Of the three PHCs, two (66.7%) had MOs and laboratory technicians in place. However, there was a significant shortfall of community health officers (CHOs), with only 58.8% of SHC-HWCs having a CHO present, indicating a 41.2% vacancy rate. The majority of SHC-HWCs had auxiliary nurse midwives in position (16; 94.1%), and over three-fourths (13; 76.5%) had multi-purpose health workers. Accredited social health activist (ASHA) workers were present at all centers (100%).

**Figure 1:**
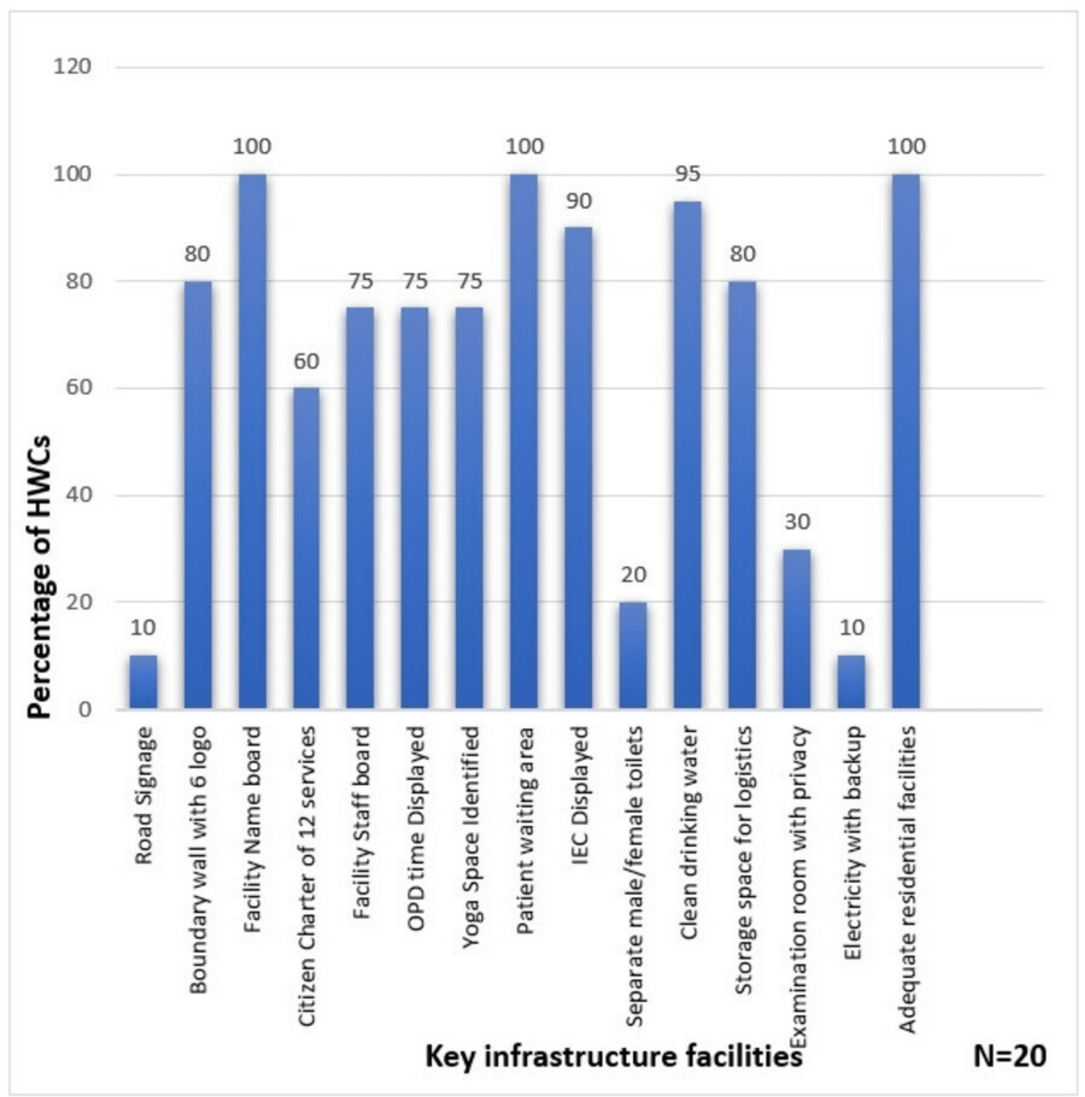
Distribution of HWCs according to key infrastructure facilities HWCs, Health and Wellness Centers; IEC, information, education, and communication

The distribution of HWCs according to key infrastructure facilities is illustrated in Figure 1.

None of the centers had the complete set of equipment and essential medicines as outlined in the operational guidelines. Only three (17.6%) SHC-HWCs had the recommended diagnostics available, and none of the PHCs met the diagnostic standards. The availability of information technology and teleconsultation facilities across HWCs is summarized in Table 1.

**Table 1:** Availability of information technology and teleconsultation facilities at HWCs (n = 20) HWCs, Health and Wellness Centers

Information technology and teleconsultation facilities	No. of centers	Percentage (%)
Internet connectivity	4	20
Desktop/laptop	10	50
Tablets	20	100
Web camera facility	10	50
Teleconsultation initiated	14	70
Teleconsultation conducted (in the previous month)	12	60

The majority of centers - 17 (85%) - had initiated yoga sessions; however, two of these (12%) had not conducted any sessions in the month preceding the visit.

As part of the Ayushman Bharat initiative, a new component was introduced involving the creation and maintenance of a population database. This includes recording details of families and individuals within the catchment area to facilitate service delivery and raise awareness about available healthcare services at HWCs.

**Figure 2:**
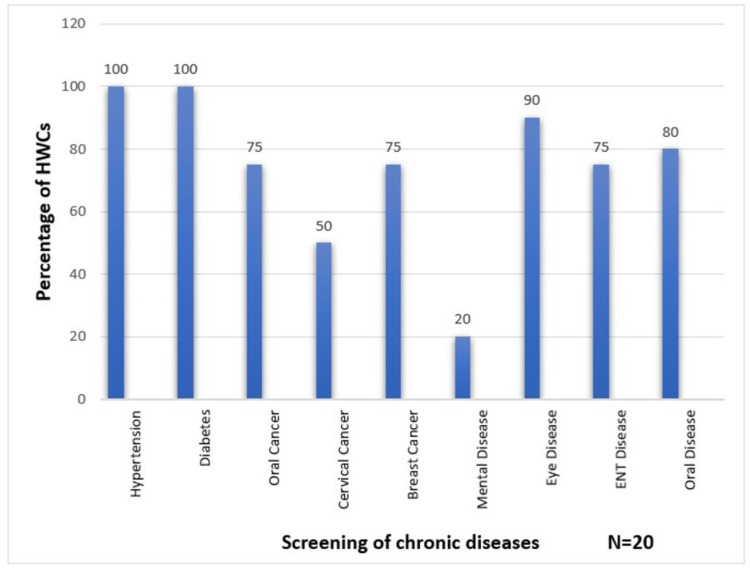
Screening of various chronic diseases at HWCs HWCs, Health and Wellness Centers

Table 2 presents the population enumeration and screening activities conducted at SHC-HWCs. Screening for chronic conditions - particularly NCDs - forms a key component of the Ayushman Bharat program. Figure 2 illustrates the range and coverage of chronic disease screenings performed at HWCs.

**Table 2:** Population enumeration and screening conducted at SHC-HWCs (n = 17) ASHA, accredited social health activist; CBAC, Community-Based Assessment Checklist; SHC-HWCs, Sub-Health Center-Health and Wellness Centers

Population enumeration and screening activities	No. of centers	Percentage (%)
Population enumeration started	14	82.4
Family folder maintained	12	70.6
Population-based screening initiated	16	94.1
CBAC form available	16	94.1
CBAC form filled by ASHA	10	58.8
CBAC recordkeeping started	17	100

The performance of the Health and Wellness Sub-Centers was evaluated based on the indicators and scoring system outlined in the Ayushman Bharat Program (Appendix A). Among the 17 SHC-HWCs included in this study, seven (41.2%) achieved a Grade B, followed by four centers (23.5%) each receiving Grades C and D. Only two centers (11.8%) achieved a Grade A, while none received a Grade E.

For further analysis, SHC-HWCs were categorized into two performance groups based on their grades. Centers with Grades A and B (scores above 75) were classified as high-performing, while those with Grades C, D, or E (scores ≤75) were categorized as low-performing. Based on this classification, 52.9% of SHC-HWCs were high-performing, and 47.1% were low-performing.

Univariate analysis revealed that SHC-HWC performance was significantly associated with the presence of a CHO at the center (p < 0.001), availability of a laptop/desktop and web camera (p = 0.050), and access to teleconsultation services (p = 0.002). Table 3 illustrates the association between performance and the input-output indicators at SHC-HWCs.

**Table 3:** Association between performance and inputs-outputs of SHC-HWCs p ≤ 0.05 was considered statistically significant. CHO, community health officer; HWCs, Health and Wellness Centers; SHC-HWCs, Sub-Health Center-Health and Wellness Centers

Inputs-outputs of SHC-HWCs	Low-performing HWCs (n = 8)	High-performing HWCs (n = 9)	Total (n = 17)	p-value
CHO				
Yes	1 (12.5%)	9 (100%)	10 (58.8%)	<0.001
No	7 (87.5%)	0 (0%)	7 (41.2%)	
Diagnostics as per guidelines				
Yes	1 (12.5%)	2 (22.2%)	3 (17.6%)	1.000
No	7 (87.5%)	7 (77.8%)	14 (82.4%)	
Laptop/tablet				
Yes	1 (12.5%)	6 (66.7%)	7 (41.2%)	0.050
No	7 (87.5%)	3 (33.3%)	10 (58.8%)	
Web camera facility				
Yes	1 (12.5%)	6 (66.7%)	7 (41.2%)	0.050
No	7 (87.5%)	3 (33.3%)	10 (58.8%)	
Teleconsultation				
Yes	2 (25.0%)	9 (100%)	11 (64.7%)	0.002
No	6 (75.0%)	0 (0%)	6 (35.3%)	
Family folder				
Yes	6 (75.0%)	6 (66.7%)	12 (70.6%)	1.000
No	2 (25.0%)	3 (33.3%)	5 (29.4%)	
Population-based screening				
Yes	7 (87.5%)	9 (100%)	16 (94.1%)	0.471
No	1 (12.5%)	0 (0%)	1 (5.9%)	

## Discussion

In the present study, a substantial number of centers performed well according to predefined indicators; however, significant gaps were also identified in human resources, infrastructure, logistics, telemedicine usage, and health promotion and screening activities. These findings are consistent with several other studies evaluating the functioning of HWCs across India.

One of the primary challenges highlighted was the shortage of human resources, particularly CHOs, who play a pivotal role in the effective functioning of HWCs. Similar observations have been made in other states. A study from Jammu and Kashmir reported that only 71% of HWCs had adequate staffing [8], while a study from West Bengal found that none of the sub-centers had CHOs. Conversely, a study in Bhopal showed that 92.4% of CHOs were present in Madhya Pradesh’s SHC-HWCs, although shortages in other cadres persisted [9]. A study from Bhubaneswar also reported that only 50% of staff nurses and lady health visitors were available [10]. These gaps in human resources pose a significant barrier to delivering primary healthcare services, especially in rural and underserved areas.

The study also identified critical issues in infrastructure and logistics, notably the lack of essential medicines and diagnostic tools, which affected the overall functionality of many HWCs. Infrastructure deficits - such as inadequate staff accommodation and unreliable water supply - were similarly reported in a study from West Bengal [11]. A report from Chhattisgarh revealed that about one-fourth of centers lacked medications for hypertension and diabetes [12], while a study from Punjab found that 50% of HWCs did not have glucometers or other essential testing supplies [13]. These deficiencies restrict healthcare providers’ ability to deliver quality care and impede the implementation of preventive and chronic disease management programs.

Although telemedicine is considered a key strategy to overcome geographical barriers and limited healthcare access in remote regions, our study found that utilization remained suboptimal despite the availability of facilities in many centers. This trend has been echoed in studies from Bhopal and Himachal Pradesh, where most sub-centers had functional laptops, but actual use of teleconsultation services was minimal [9,14]. This disparity suggests that while infrastructure may be in place, barriers such as inadequate training, low digital literacy, and unreliable internet connectivity hinder full utilization. Furthermore, studies from Jammu and Kashmir and Western Odisha highlighted a disparity between urban and rural areas, with urban PHCs showing significantly higher teleconsultation usage (88%) compared to rural PHCs (60%) [8,15]. Bridging this digital divide will require investment in digital training, improved internet infrastructure, and equitable access to telemedicine tools across all settings.

Health promotion and NCD screening activities were widely implemented across HWCs, aligning with national goals to promote early detection and healthy lifestyles. In our study, 85% of centers had initiated yoga sessions, substantially higher than the 58.3% reported in a study from Bhubaneswar [10]. This variation may be attributed to differences in methodology, policy implementation, regional focus, or operational efficiency. The inclusion of yoga reflects growing recognition of its role in promoting both physical and mental health at the community level.

Although all centers had implemented screening programs for hypertension and diabetes, cancer and mental health screenings were less frequently conducted. A similar study from Bhopal reported that while most HWCs had initiated NCD screenings, cancer screenings were rare, and mental health and ophthalmic screenings were seldom performed [9]. In contrast, a study from Jammu and Kashmir found even lower detection rates, with only 6% of hypertensive and 4% of diabetic patients being identified [8]. Although our study did not explore the specific causes behind regional differences, the low rates of cancer and mental health screenings suggest systemic challenges - such as limited availability of specialized personnel, insufficient training, or lack of prioritization - that likely vary across geographic regions. These findings highlight the need for a more comprehensive screening strategy that includes cancer, mental health, and ophthalmic conditions.

Strengths and limitations

This study’s strengths include its policy relevance, systematic sampling methodology, and use of performance indicators aligned with national guidelines, which enhance its practical utility. However, the findings are limited to a single block in Dhamtari District, Chhattisgarh, and may not be generalizable to the entire state. As Ayushman Bharat was launched in 2018 and most evaluation studies emerged post-2021, comparable literature remains limited. Although there is a possibility of observer bias, steps were taken to minimize this through pilot testing and standardized training in data collection procedures.

## Conclusions

To maximize the contribution of HWCs in achieving comprehensive primary healthcare, a more holistic approach is essential. This includes addressing human resource needs, ensuring logistics and infrastructure as per operational guidelines, providing uninterrupted teleconsultation services, enhancing coverage of information, education, and communication activities, and expanding screening services. All HWCs should strive toward improved performance to meet the objectives of the Ayushman Bharat initiative.

## Appendices

Appendix A 

**Table 4:** Indicators for assessing the performance of SHC-HWCs Source: [5]

Sl no.	Indicators	Score 2	Score 3	Score 4	Score 5
1	Number of OPD cases in the month	250 per month for 5000 population or 150 per month for 3000 population	300 per month for 5000 population or 180 per month for 3000 population	250 per month for 5000 population or 210 per month for 3000 population	400 per month for 5000 population or 240 per month for 3000 population
2	Proportion of estimated pregnancies registered	At least 50% of the estimated pregnancies	At least 60% of the estimated pregnancies	At least 80% of the estimated pregnancies	At least 100% of the estimated pregnancies
3	Proportion of pregnant women registered who received ANC	At least 50% of the pregnant women received ANC as per schedule	At least 60% of the pregnant women received ANC as per schedule	At least 80% of the pregnant women received ANC as per schedule	At least 100% of the pregnant women received ANC as per schedule
4	Proportion of children up to 2 years of age who received immunization	At least 50% of the children received immunization as per schedule	At least 60% of the children received immunization as per schedule	At least 80% of the children received immunization as per schedule	At least 100% of the children received immunization as per schedule
5	Proportion of newborns who received HBNC visits	At least 50% of newborn received HBNC visits	At least 60% of newborn received HBNC visits	At least 80% of newborn received HBNC visits	At least 100% of newborn received HBNC visits
6	Proportion of above 30 years individuals screened for hypertension	At least 2% screening of individuals over 30 years	At least 4% screening of individuals over 30 years	At least 6% screening of individuals over 30 years	At least 8% screening of individuals over 30 years
7	Proportion of above 30 years individuals screened for diabetes	At least 2% screening of individuals over 30 years	At least 4% screening of individuals over 30 years	At least 6% screening of individuals over 30 years	At least 8% screening of individuals over 30 years
8	Proportion of above 30 years individuals screened for oral cancer	At least 2% screening of individuals over 30 years	At least 4% screening of individuals over 30 years	At least 6% screening of individuals over 30 years	At least 8% screening of individuals over 30 years
Sl no.	Indicators	Score 2	Score 3	Score 4	Score 5
9	Proportion of patient of HTN on treatment	40% of patients who received treatment	60% of patients who received treatment	80% of patients who received treatment	100% of patients who received treatment
10	Proportion of patient of DM on treatment	40% of patients who received treatment	60% of patients who received treatment	80% of patients who received treatment	100% of patients who received treatment
11	Proportion of cases referred for TB screening	Minimum 2% cases identified from OPD should have been referred for screening of TB at a higher facility		Minimum 3% cases identified from OPD should have been referred for screening of TB at a higher facility	
12	Notified TB patients who received treatment as per protocols8	100% of patients on treatment			
13	VHND held against planned	MPWs and ASHAs will organize all VHND session as planned and CHO should monitor at least two VHNDs in a month for performance- linked incentive			
14	Village meetings (VHSNCs)/MAS held	MPWs and ASHAs will organize all VHNC session as planned and CHO should monitor at least two VHNSC meeting in a month for performance- linked incentive			
15	Monthly meetings held at SHC- HWCs organized monthly meeting with primary care team at sub-centers	One meeting held at the SHC- HWC and should be attended by MPWs and all ASHAs			
16	HWC portal daily reporting status	20 days or more	22 days or more	24 days or more	26 days or more
17	HWC portal monthly reporting status	Before 8^th^ of every month	Before 7^th^ of every month	Before 6^th^ of every month	Before 5^th^ of every month
18	Telemedicine services	At least 4 consultations	At least 6 consultations	At least 8 consultations	At least 10 consultations
19	Conduction of wellness activities	At least 8 activities per month	At least 10 activities per month	At least 12 activities per month	At least 14 activities per month
20	CBAC form entry	At least 50% of target achieved	At least 60% of target achieved	At least 80% of target achieved	At least 100% of target achieved
		HWCs completed CBAC form entry given score 5			
